# Clarifying concepts: “Well-being” in sport

**DOI:** 10.3389/fspor.2023.1256490

**Published:** 2023-11-28

**Authors:** Lisa Raquel Trainor, Andrea Bundon

**Affiliations:** School of Kinesiology, The University of British Columbia, Vancouver, BC, Canada

**Keywords:** eudamonia, eudaimonic well-being, hedonia, hedonic well-being, psychological wellbeing, subjective wellbeing, athlete well-being

## Abstract

The purpose of this commentary is to critique the application of well-being in the field of sport and exercise psychology and to provide recommendations for future research. Over the last decade well-being has been an increasingly popular concept under investigation. In the field of sport and exercise psychology, numerous scholars have examined and conducted research on well-being of athletes. While this research has resulted in an abundance of findings, there is concern in how the concept of well-being was applied, defined, and measured. The construct of well-being can be traced back to two distinct perspectives, hedonic well-being and eudaimonic well-being. These perspectives of well-being are based on different philosophical assumptions, and while they are compatible, they are theoretically distinct. In sport and exercise psychology, well-being has lacked consistent operationalization and measurement (i.e., theoretical alignment, single dimensions of hedonic or eudaimonic measured to make claims about the broader well-being constructs), is vague and loosely defined, and is often studied in isolation from a well-being perspective (i.e., no theoretical foundation). We conclude by offering three recommendations to move the field of well-being in sport research forward.

## Introduction

Well-being has become an increasingly popular area of study in sport and exercise psychology in recent years (1–12). Over the past decade there has been a shift from exploring the presence of psychopathy to the existence of wellness, well-being, and psychological growth (13). Well-being is more than the absence of problems and disease (14, 15), and within the literature has been described and studied in a multitude of ways. The purpose of this paper is to critique the current application of well-being in the field of sport and exercise psychology, and to bring awareness regarding future applications of the concept of well-being. First, we provide a brief overview of the perspectives of well-being, connections to mental health, and the importance of athlete well-being, followed by well-being research in the context of sport and exercise psychology (application and limitations), and conclude with recommendations to move the field forward.

### Well-being

The concept of happiness has long been at the center of many arguments surrounding “*what is well-being”*. Well-being is a “complex construct rooted in health, philosophy, and psychological practices” (16, p. 206). Well-being *in general* is defined as, “optimal psychological experience and functioning” (13). While this definition provides a starting point to study well-being, it is too broad and fails to ascertain what is “optimal” experience and function. Historically, well-being has been separated into two distinct perspectives of well-being, hedonism/hedonic well-being (HWB) (17) and eudaimonism/eudaimonic well-being (EWB) (18). Both perspectives address different features of what it means to be well (19). Tatarkiewicz, a philosopher, defined hedonism (subjective happiness) as a happy life that was enjoyable, not due to what one had, but the pleasant reaction to life circumstances (17). Socrates, Plato, and Aristotle formulated the idea of eudaimonism, where happiness was associated with “flourishing”, possessing the greatest goods, and “the good life” (20, 21). However, the conceptualization of well-being has been debated in the field of psychology, resulting in a number of definitions derived from different conceptual and theoretical frameworks (22–25). The discrepancy in the operationalization of HWB and EWB has resulted in perspectives being used inconsistently and interchangeably (22).

### Hedonic well-being

Hedonic well-being (synonymous with subjective well-being and emotional well-being) (26–28) includes one's affective and cognitive evaluations of their lives (29, 30). “Subjective” well-being implies that each individual assesses the degree to which they feel well (29). Thus, within hedonism people are to decide the degree to which they are satisfied based on their personal values, goals, and life circumstances (31). Hedonic well-being is defined as “experience[ing] a high level of positive affect, a low level of negative affect, and a high degree of satisfaction with one's life” (13). The three main components of HWB are positive affect, negative affect, and life satisfaction. Positive affect refers to pleasant moods and emotions, such as joy and excitement. Positive emotions are a component of HWB because they reflect one's assessment that life is proceeding favourably (13, 14, 32). Negative affect refers to unpleasant moods and emotions, such as anxiety and anger, and are a component of HWB because they reflect one's negative assessment of life, health, and events (13, 14, 32). Life satisfaction refers to the cognitive aspect of HWB; one's judgments of personally significant life domains, such as family, work, leisure, and mental and physical health (13, 14, 32). One assesses their current satisfaction with each of their major life areas and evaluates how close to ideal these are (13, 14, 32). The definition of HWB is (for the most part) agreed upon by scholars.

### Eudaimonic well-being

Eudaimonic well-being (synonymous with psychological well-being; PWB) (33–36) relates to fulfillment, positive functioning, and cultivating the greatest good (20). This perspective is concerned with how one grows from life challenges to live a life that is aligned with personal goals and values (24, 37, 38). Broadly speaking, EWB has been defined as “living well or actualizing one's human potentials” (13). A number of researchers believed there was more to happiness than positive affect and a sense of satisfaction (i.e., HWB) (19, 21, 34, 39), and research has indicated that eudaimonia is a necessary factor contributing to the “good life” (31). Eudaimonia is more than feeling that one's life has meaning, it is a matter of cultivating higher purpose and complexity (20, 40). After exploring the multiple conceptualizations of EWB in the literature, the most prevalent contents of EWB include meaning, personal growth/self-realization, excellence, and authenticity/autonomy/integration. Of these core concepts of eudaimonia, meaning in life is one of the most dominant in definitions across research (22, 41, 42). Eudaimonia is not an outcome, rather it is a way of living, living in accordance with one's daimon and fulfilling one's potential (20). Huta and Ryan (39) found that EWB activities were more related to meaning and long-term benefits and HWB activities were more related to positive affect and short-term benefits. The existential aspects of EWB (i.e., purpose in life and personal growth) are most distinct from the affective/quality assessments of HWB (i.e., pleasure and satisfaction) (19). For example, eudaimonic activities are not always pleasurable (i.e., low positive affect) and can be challenging, yet purposeful and meaningful to the individual. Eudaimonism and hedonism are strongly related and experienced simultaneously (41), but are “distinct conceptions of well-being” (19).

### Multiple conceptualizations of eudaimonic well-being

Presently, there are at least 45 different ways to operationalize EWB and at least 63 constructs used to measure EWB (42). There is also no agreement on the core elements that comprise EWB. Unfortunately, this makes research on EWB essentially incomparable (22, 41–43). There are a number of researchers who have formulated different (leading) conceptualizations of EWB. These researchers included: Alan Waterman, Carol Ryff, Corey Keyes, Richard Ryan and Edward Deci, Veronika Huta, Jack Bauer, Martin Seligman, Joar Vitterso, Michael Steger, Antonella Della Fava, Blaine Fowers, Frank Martela and Kennon Sheldon, Felicia Huppert and Timothy So, and Ed Diener. All of these researchers have contributed and provided the basis of our understanding of EWB today and their works formulates the basis for most EWB studies. While multiple conceptualizations of a concept are not problematic, it is problematic if the concept is atheoretical applied. For example, researchers state they are studying EWB (or fail to identify a well-being perspective), but measure positive and negative affect and subjective vitality, and then make claims in regard to EWB. In practice well-being is more often than not atheoretically applied. We have provided a table below to summarize the most common conceptualizations of EWB in the literature, outline the researcher's definition and core concepts of EWB (Table 1).

**Table 1:** Conceptualizations of eudaimonic well-being.

Founder	EWB Definition	EWB Key Concepts
Alan Waterman (38), Waterman et al. (36)	Quality of life as a result of developing personal potential, fulfillment of personally expressive self-concordant goals.	• Self-discovery • Perceived development of one's true self • Sense of purpose and meaning • Significant effort in pursuit of excellence • Enjoyment of activities (flow) • Personally expressive activities
Carol Ryff (34)	Three principles underlie positive well-being: (1) an articulation of the good life; (2) mental and physical components; and (3) viewed as a multidimensional process rather than an end state.	• Self-acceptance • Autonomy • Positive relations • Environmental mastery • Purpose in life • Personal growth
Corey Keyes (14)	Eudaimonia as a combination of Ryff's PWB and social well-being. Keyes’ model of flourishing is a combination of hedonic (emotional), eudaimonic, and social well-being. This was later developed into dual continua model of mental health (languishing to flourishing and floundering to struggling).	• Social acceptance • Social actualization • Social contribution • Social coherence • Social integration
Martin Seligman (44)	Working toward self-flourishing. Identifying personal virtues and strengths and developing them for social contribution and service.	• Positive emotions • Engagement • Relationships • Meaning • Accomplishment
Richard Ryan and Edward Deci (24)	Fulfillment of the basic needs is required for psychological growth and development, and fulfillment of the three basic needs is a process that represents eudaimonic living.	• Autonomy • Competence • Relatedness
Frank Martela and Kennon Sheldon (42)	Includes aspects of both “feeling well” and ‘doing well’.	• Eudaimonic motives and activities (values, motives, goals, and practices) • Psychological needs satisfaction (autonomy, competence, and relatedness)
Veronika Huta (45)	Utilizing and developing one's best self (i.e., self-actualization) in accordance with one's deeply held values (i.e., universal nature fulfillment).	• Meaning • Elevating experiences • Self-connectedness
Jack Bauer (46)	Personal narrative toward personal growth through ego development and maturity over time.	• Pleasure • Meaningfulness • A deep self-understanding
Joar Vitterso (47, 48)	Main function of EWB (interest and engagement) is to promote mental and physical growth.	• Preference for complexity • Curiosity • Engagement/flow • Personal growth • Competence • Meaning and purpose in life • Self-actualization • Excellence
Antonella Della Fave (49)	An emphasis on cultural context to determine the ‘good life’ and optimal experiences (examining individual and social level).	• Flow (optimal experience) • Long-term meaning making
Blaine Fowers (50)	EWB is engaging in activities that are personally worthy, meaningful, aligned with vales and self-identity.	• Flourishing • Pursuing excellence • Virtue • Meaningful relationships
Michael Steger (51)	Eudaimonia consist of self-expressions that are consistent with one's goals, values, relationships, self-development, and self-improvement.	• Eudaimonic behaviours (brings personal meaning; related to values, autonomy, goals, purpose, social responsiveness, and self-development and improvement)
Felicia Huppert and Timothy So (52)	Flourishing as a concept to capture positive feeling (hedonic) and positive functioning (eudaimonia) and positive mental health.	• Competence • Emotional stability • Engagement • Meaning • Optimism • Positive emotion • Positive relationships • Resilience • Self-esteem • Vitality
Ed Diener (53)	Proposed the term ‘flourishing’ to account for hedonic and eudaimonic well-being.	• Purpose in life • Positive relationships • Engagement • Competence • Self-esteem • Optimism • Contribution towards others’ well-being

### Well-being and mental health

It is agreed that while HWB and EWB are philosophically distinct, they share substantial overlap (13, 19, 36, 54) and complement each other as they ask different questions of what it means to be “well” (i.e., positive feeling vs. positive functioning) (24). Those who pursue both HWB and EWB have more well-rounded wellbeing, experience both immediate well-being (e.g., through hedonic activities) and delayed benefits of well-being (e.g., through eudaimonic activities) (45, 55). Further, Keyes (14) has argued that both HWB and EWB play important role in contributing to mental health (14).

Hedonic, eudaimonic, and social well-being are all important dimensions of mental health (14). This means that one has positive feelings (hedonic), positive functioning (eudaimonic), and is contributing to society (social) (14, 56). Keyes (14) defines complete mental health and high well-being as flourishing, which means being “filled with positive emotion and to be functioning well psychologically and socially” (p. 210). When individuals have high and equal levels of both HWB and EWB they may experience a sense of congruency (19), and have higher levels of mental health (14). On the other hand, Keyes (14) defines incomplete mental health as languishing with low well-being, associated with an emptiness, stagnation, and despair, similar to depression. However, the absence of mental illness does not mean the presence of mental health, nor does the presence of well-being mean the absence of mental illness. This is captured by the dual continua model of mental health and well-being which postulates that mental health and mental illness are related but distinct concepts (14). For example, one can have positive mental health and experience mental illness, or one could have low mental health without a mental illness. In contrast to mental health, optimal psychological functioning is generally understood as “functioning well” (cognitively) *and “*doing well” (affectively) (42). Most researchers agree that both hedonia and eudaimonia are needed for optimal psychological functioning, flourishing, and the good life (14, 24, 39, 44, 57). Thus, mental health, mental illness, and well-being are distinct concepts that converge to form different combinations of struggling, floundering, languishing, and flourishing (14, 15, 56, 58).

### Current challenges

Well-being remains an ambiguous term lacking clear conceptualization within the literature (24, 59). This is largely due to the lack of theoretical rationale for conceptualizations of well-being (34). There is great inconsistency in how EWB is studied; it lacks consistent operationalization and measurement (i.e., theoretical alignment, single item dimensions capturing only one component of the well-being perspective to make claims about the broader well-being construct), is *vague* and *loosely* defined, and is often studied in isolation from a well-being perspective (i.e., eudaimonia or hedonia) (22, 42, 60). The lack of understanding of EWB across studies poses significant challenges when attempting to consolidate and synthesize research findings. Research on EWB is reaching a point where the inconsistency in how EWB is studied renders findings amongst studies somewhat incomparable, stagnating our understanding of EWB. We are not arguing that there needs to be one definition of EWB, instead greater articulation and clarity into how researchers are investigating “well-being”.

### Athlete well-being

It is clear that elite sport has the potential to benefit and disrupt the EWB of athletes (61). Research has found that athlete well-being is associated with athlete success; “successful” athletes often report an ability to cope with anxiety, be mentally resilient, and have strong support networks (62). While elite sport can support athlete well-being it can also disrupt athlete well-being, leading to mental health issues (9, 63–65). Elite sport is physically and mentally demanding (47), where the increased stress from elite sport makes athletes more susceptible to poor mental health (64, 66). Research has supported the claim that elite athletes are more likely to experience poor mental health in comparison to the general population (64, 67, 68). This is concerning because elite athletes are also less likely to recognize, acknowledge, and seek support for their mental health (68). This is likely underpinned by stigma, lack of understanding, and perceived influence of weakness and performance implications (67, 69, 70).

Despite the increased interest and prevalence of mental health concerns among elite athletes, research has yet to identify that factors that influence athlete well-being and mental health (5), meaning that there is little insight into how to best support athlete well-being. Furthermore, while there have been efforts to examine the prevention, identification, and early treatment of athlete mental health, sport governing bodies continue to dismiss the significance of athlete well-being more broadly (70). It is vital to understand athlete EWB, as disruptions to EWB is a predictor to future risk of mental illness (71). Currently, there are no models of care to support athlete well-being and mental health (63).

## Discussion

In sport, well-being has been studied in various populations, for example coach and practitioner well-being (72–76) and organizational well-being (77–79). However, for the purpose of this commentary and critique, we will focus on athlete well-being. After such a heavy focus on physical health, there has been a growing interest in athlete well-being (1–12, 61, 64, 67, 68, 80–83). There has been an increased interest in athlete well-being as a number of high-profile athletes have announced distress in sport (i.e., Michael Phelps, Simone Biles, and Naomi Osaka), and widespread concerns of abuse in sport, which have raised trepidations over the duty of care toward athletes.

Research has indicated that athlete well-being can contribute to improving health and development (83). Participation in sport can foster physical, social, and psychological benefits (85, 86) and contribute to well-being (87). However due to the extreme physical and mental demands, negative experiences in sport are not uncommon (64, 66, 85). The pressures of elite sport can leave athletes vulnerable to psychological disruptions (9, 63, 88, 89). For this reason, not all physical activity promotes EWB (90). Elite sport elicits increased psychological challenges such as internal and external pressures, transitions, and injuries (10, 91, 92). Elite athletes may be at greater risk for disruptions to well-being (93) and psychological health (67, 68). These results along with the duty of care toward athletes demonstrate the importance of athlete well-being, signifying the need for proper application, coherent measurement, and clear reporting on the concept of well-being.

### Limitations of well-being in sport

Despite the conceptual ambiguity of EWB, researchers have still attempted to study well-being in specific contexts (i.e., sport). This research has covered a broad range of topics including examining tenets of self-determination theory, achievement goals, coach interpersonal styles, cognitive appraisals, passion, high-performance narratives, and injury (1–12, 61, 67, 68, 81, 82, 94–106). Despite current research into athlete well-being, and some advances, there have been a number of limitations. Studies have failed to identify the perspective of well-being under investigation, operated atheoretically or “theoretically confused” (i.e., mixing and matching hedonic and eudaimonic perspectives), used a “random assortment” of measures, employed deductive methods, and have failed to distinguish between *global* well-being (overall well-being) and *sport-specific* well-being (contextualized well-being). Very few studies have explored sport-specific *EWB*, limiting definitions of athlete EWB to previously conceptualized general (or global) frameworks of well-being. Thus, we believe no conclusive claims can be made in regard to *athlete* EWB in the sport context.

Some previous research in the area of well-being in sport has confused well-being perspective (defined EWB as HWB), defined well-being as solely the absence of psychological distress (i.e., measured depression to claim the presence or absence of well-being), and used single dimensions to measure well-being (i.e., positive and negative affect scale) (107, 108), to make claims related to HWB or EWB. The presence of psychological health should not be limited to absence of psychological disturbances, but it should include positive psychological indicators such as positive affect, purpose in life, growth and development, and social impact (25, 109, 110). Eudaimonic well-being cannot be reduced to a measurement of a single dimension, as many theoretical conceptualizations of EWB outline multiple facets of positive functioning (24, 34, 38, 44, 45, 111, 112). More recently, researchers have shifted to use a number of indicators to measure well-being such as life satisfaction, affect, subjective vitality, psychological needs, self-esteem, and psychological distress (85). Subjective vitality has been the most commonly used indicator of one's EWB and has been identified as a characteristic of one experiencing EWB (24), but only captures one dimension of EWB. Although these indicators are necessary to understand well-being, individually they are insufficient to provide a complete representation of well-being (85), and cannot be used to make claims to the broader EWB construct. When this happens, it results in a compromised understanding of athlete EWB. On the other hand, in cases where researchers are using a number of indicators to measure well-being, they are not theoretically aligned with one perspective of well-being (hedonia or eudaimonia), yet claims and inferences about HWB and EWB are being made. Thus, limited claims can be made about the *eudaimonic* well-being of athletes.

After reviewing the literature, most studies clearly operate from “confused” or “blended” well-being perspectives, “mixing and matching” *only some* concepts from both hedonic and eudaimonic perspectives. While it is acceptable to study one dimension of HWB or EWB, it is problematic when the single dimension is used to make claims to the broader HWB or EWB construct. Further, the overwhelming majority of researchers *do not theoretically position* their use of the concept of well-being; instead, they just study “well-being”. This results in an incoherent exploration of well-being, using some measure such as the positive and negative affect scale (113) and the satisfaction with life scale (114) to study concepts that theoretically align with HWB, *and* the subjective vitality scale (115) to study concepts that theoretically align with EWB. Alone, these measures are not sufficient to gain complete insight of HWB or EWB. In this case, claims can only be made about positive and negative affect, satisfaction with life, and vitality. While claiming to explore “athlete well-being”, they fail to identify a well-being perspective or study the “complete” well-being concept. Even though well-being has not been theoretically justified in the majority of studies, we conclude that HWB has been the primarily adopted point of view. Positive and negative affect has been the most employed measure(s) to examine well-being; while this measurement is successful in capturing the affective dimension of wellbeing (HWB) it fails to examine the other dimensions of psychological functioning (EWB) (116). While some researchers have clearly defined the theoretical construct of well-being under investigation, the measurements used to assess well-being have not always aligned with said theoretical perspective. Essentially these results tell us little about well-being, as well-being is theoretically fused to hedonia or eudaimonia. Well-being has been “treated as a substantially unspecific variable, is inconsistently defined, and is assessed using a variety of theoretically questionable indicators” (117).

The differing philosophical perspectives (hedonia and eudaimonia) of well-being lead to different ways of examination regarding the causes, consequences, and dynamics of well-being (24), and ultimately inconclusive results when looking across the well-being in sport literature. Lundqvist (117) argues that researchers need to explicitly state which perspective they are operating from and if utilizing quantitative methods need to *use established measurements that theoretically fit* with the chosen well-being perspective. Due to conceptual ambiguity and inconsistent definitions and measurements between studies, it is difficult to compare results, generalize findings, or develop a theoretical base of knowledge (117). With future research clearly stating their well-being perspective, as well as working within theoretical alignment (concepts and measures), we can start to synthesize related well-being research and begin to theoretically understand athlete EWB and how to better support athlete EWB.

### Global vs. sport-specific well-being

In the field of sport psychology efforts to contextualize athlete well-being into its own theoretical framework have been rare (117). In addition to failing to define the type of well-being under examination (eudaimonic or hedonic) and using a variety of names to describe the construct (i.e., mental well-being, emotional well-being, well-being) without clarification, past research has failed to distinguish between *global* well-being (overall well-being) and *sport-specific* well-being (contextualized well-being) (e.g., (118, 119)). Global well-being refers to “contextual-free subjective evaluations of a person's life” (117). Whereas sport-specific well-being is characterized by attending to context specific domains (117). Currently, there is no sport-specific measure of EWB (118). The only way to assess athlete EWB (sport-specific EWB) at this time is to deductively measure global EWB by separately measuring each dimension of EWB (118). For example, the six dimensions of Ryff's (34) model (autonomy, environmental mastery, self-acceptance, positive relations, purpose in life, and personal growth) or Seligman's (44) model (positive emotion, engagement, relationships, meaning, and accomplishment). For example, using Ryff's (34) model in sport research would include examining *self-acceptance as an athlete, positive relations in sport (i.e., teammates, coaches, etc.), autonomy in sport, sport environmental mastery, purpose in sport,* and *personal growth as an athlete.* Whereas, using Seligman's (44) model in sport research would include examining *positive emotions in sport, engagement in sport (environment, commitment), relationships in sport (i.e., teammates, coaches), meaning in sport (attachment, emotions), accomplishment in sport.* The reliance on global measures of well-being will not result in a complete understanding of an individual's well-being (117). These assessments are *not ideal*, and results lack clarity in understanding the complex relationship between athlete EWB and sport (118). It is also unknown if the previously formulated theories of (global) overall EWB (14, 34, 44) are even applicable to the sport context (i.e., sport-specific EWB). As these global theories were developed in non-sport contexts, they do not take into account the unique psychosocial challenges presented in the sport context (discussed below), there may be different (and significant) components that comprise athlete EWB that are overlooked when deductively examining athlete EWB with previously formulated global theories of EWB.

Very few studies have explored sport-specific EWB from a fully eudaimonic perspective (i.e., explored all eudaimonic concepts in a single model) and the handful that have, utilized deductive methods (96, 120, 121). While this is a *very important first step* in developing understanding around athlete EWB it limits definitions of athlete EWB to previously conceptualized global EWB frameworks rather than exploring athletes' perceptions of EWB in the sport context (contextual; sport-specific EWB). Future research needs to move away from deductive methods to inductive (or abductive) methods to understand (contextualized) athlete EWB. Three qualitative studies have explored EWB in sport. Both Ferguson et al. (120) and Lundqvist and Sandin (96) used Ryff's (34) model of EWB to deductively investigate athlete EWB, where Mirehie and Gibson (121) applied the PERMA model (44) to deductively examine well-being in a sample of recreational snow sport athletes. These studies merely explored how Ryff's (34) and Seligman's (44) model could be applied to the sport context. While the studies provide some insight on EWB in the sport context, they failed to explore other contextually relevant dimensions of EWB due to its deductive nature. For example, Ryff (34) or Seligman (44) models (or any other EWB model) could be applicable in sport to an extent, but how do we know what *might* be contextually relevant to athletes EWB in sport if we do not use inductive or abductive methods to theorize athlete EWB? An issue, or limitation, with this research is that it does not account for athlete driven context-specific conceptualization of EWB.

Due to the limitations of sport-specific EWB, other recent studies which have explored athletes' well-being from a fully eudaimonic perspective using global frameworks of EWB to inform and guide their study design, data analysis, and resultant claims (1, 2, 7, 10–12, 61, 81). While this is theoretically aligned and situated work, results from these studies are still constricted to global frameworks of EWB and limited in the claims that can be made in regard to *sport-specific EWB.* In other words, in the field of sport psychology we are reaching a point where there is a plethora of research on athlete well-being, yet there is limited understanding of *what is sport-specific EWB*.

This also holds true for measurements of global EWB that have been adapted to sport. In the last five years measures such as the Sport Mental Health Continuum Short Form (122) adapted from Keyes (58) and the Eudaimonic Well-Being in Sport Scale (80) grounded in self-determination theory (24). While these “sport-specific” measures tick the box of studying well-being from a “fully eudaimonic” perspective, they are global measures of EWB that have been *adapted* to the sport environment. Even Kouali et al. (80) comment on their EWB in sport scale and identify, “a notable limitation of this research is the absence of a sport specific measure of EWB”. The issue is that the contextual component of sport-specific well-being is still missing. How do we know if global measures of well-being can truly capture the idiosyncrasies in the sport environment? Future research that explores contextually relevant components of sport-specific EWB is needed to gain insight, a greater understanding, and a starting point for research on athlete well-being.

### An emphasis on context

The context-specific view of well-being in sport is an important component in understanding athlete EWB, as a person's judgment of their well-being is related to personally significant contextual domains (123). This general phenomenon is especially resonant in the context of high-performance sport, as athletes dedicate vast amounts of time to sport where they have few opportunities to pursue alternative interests (124). Athletes' evaluation of their well-being “are often *exclusively grounded in their sport experience*” (117); emphasis added). Sole reliance on global well-being risks an incomplete assessment of one's well-being (125), and fails to explore what constitutes athlete EWB. Domain specific well-being assessments are necessary “to capture the subtleties, complexities, and variations of [peoples] cognitive and affective experiences” (126). Individual accounts of well-being are vital as they provide insight into the “picture of well-being that is grounded in people's preferences, rather than in a priori judgements about what should be the most important aspects of well-being” (Organization for Economic Co-operation and Development (127). Knowledge of the factors that comprise context specific well-being is needed to understand and better support athlete EWB. More consistent examinations of context specific EWB are warranted (94, 96, 117), further supporting the need for athlete driven conceptualization of sport-specific EWB.

We argue that well-being still remains an ambiguous term lacking clear conceptualization within the literature. Well-being is frequently atheoretical and “*whatever an investigator has identified* as being the psychological dependent measure in her/her investigation” (128); emphasis added). Consequently, there is *great inconsistency* in the well-being literature, and this includes the well-being of athletes.

## Moving forward

Throughout this commentary we have explored understandings of EWB from past and present to critically examine the application of well-being in the sport and exercise psychology literature. Through engagement with and synthesis of the literature, we have highlighted the conceptual opacity of EWB within sport and exercise psychology research. From no agreed upon definition of EWB, to inconsistent/amalgamated definitions, to incoherent combinations of the two philosophical traditions of well-being (hedonic and eudaimonic) through the use of various measures and elements from different theoretical approaches, EWB still remains inconspicuous and ambiguous in the sport psychology literature. It is important to understand how the concept of well-being has been applied in past literature, as it will assist researchers to critically evaluate how they define well-being and how they apply well-being. In order to advance research on well-being in sport psychology we have provided a few key areas to contemplate as a precursor to further research on well-being in sport.

### #1 operating from a distinct well-being tradition or a complete combination of perspectives

In order to move this field forward the first step is identification of the well-being tradition under investigation. For example, is there interest in exploring the “feeling” aspect of well-being (e.g., hedonia), the “functioning” aspect of well-being (e.g., eudaimonia), or both (e.g., flourishing; hedonia and eudaimonia)? While there does not need to be a singular definition of well-being, there needs to be some consistency between “type” (tradition) of well-being under investigation. For example, if the eudaimonic tradition of well-being is under investigation, how will EWB be defined in this study? What are the key dimensions that will be used to explore EWB? On the other hand, both HWB and EWB can be studied together as they are complimentary perspectives. One can use well-being frameworks that take into account both the hedonic and eudaimonic perspective (14, 53); or utilize independent models of HWB and EWB. The key takeaway is that the construct (hedonic, eudaimonic) needs to be identified and measured in complete form, that is, taking into account all the concepts and components identified in the model. Being able to identify the well-being tradition and a “definition” of well-being will aid in comparing, synthesising, and theorization of research findings. This will also likely lead to an increase in research being theoretically situated (i.e., hedonic, eudaimonic, or both), and decrease the output of “theoretically confused” and “theoretically incomplete” research.

### #2 theoretically aligning measurements of well-being to the tradition of well-being

A common trend in the research is for researchers to select a few scales in order to measure well-being. Because a perspective of well-being has not been identified it results in a mix and match approach to study well-being. From the literature on well-being, an overwhelming majority focuses on short term affective well-being (i.e., happiness) as the core of positive functioning (34). Unfortunately, this view neglects the array of concepts that capture positive functioning (i.e., purpose, ego development, relationships, self-realization) (34). Thus far, the most common indicators to measure well-being consist of variations of the Satisfaction with Life Scale (114), the Positive and Negative Affect Scale (113), and the Subjective Vitality Scale (115). Some of the concepts addressed within these scales theoretically line up to fit with HWB (Positive and negative affect, satisfaction with life) where others fit with EWB (vitality). Some of these studies even use depression and burnout scales to identify the presence or absence of well-being, even though well-being is not merely the absence of disease and illness, but rather the presence of elements of positive functioning. It has been found that ill-being and well-being are distinct concepts, rather than polar opposites (34, 129). The presence of negative emotions in psychopathology does not mean the absence of positive emotions (130).

A large portion of existing studies are operating from a “mixed-bag” approach to well-being, meaning that their well-being measures combine some hedonic elements and some eudaimonic elements. The few measurements that are used to study well-being are only capturing limited components of the different perspectives of well-being (hedonic and eudaimonic), which renders findings inconclusive to claims on either hedonic or eudaimonic well-being. For example, it is agreed upon by researchers that HWB is composed of three dimensions, *positive affect, negative affect, and life satisfaction* (14, 24, 34, 38). Each of these three dimensions need to be measured, in order to fully study HWB and make claims about HWB. Whereas there are a number of conceptualizations of EWB, but according to Ryff (34) it is composed of six dimensions, *autonomy, environmental mastery, self-acceptance, positive relations, purpose in life, and personal growth*, which all need to be measured to study global EWB. Researchers need ask, what perspective of well-being is under investigation? What are the key concepts that need to be measured or discussed? What claims can I make in regard to hedonic or eudaimonic well-being? The multiple and incomplete implementation of well-being measurements have led to different conclusions, insights, and recommendations of well-being in sport. Researchers need to explicitly state which perspective they are operating from and use established measurements that theoretically fit with the chosen perspective. The use of global and aligned measure of well-being will allow for comparisons between studies that could offer general insights into how athletes are psychologically functioning and feeling. Further, the continued use of global measures can be used to identify which components of global well-being are relevant to the sport context while also leaving room to consider what may still be missing.

### #3 employing qualitative research to identify context specific dimensions of well-being

While global frameworks of well-being allow for some insight into athlete well-being, concurrent attention needs to be placed on the investigation of context specific EWB. Context specific measures of EWB can help identify *what is* missing from global frameworks. It is very likely that there are sport-specific components of athlete well-being that are missed when utilizing global frameworks. While it is expected that global frameworks of EWB will share overlap with sport-specific frameworks, it is also expected that there will be contextualized components of sport-specific well-being as sport is situated in a unique sociocultural environment.

In order to draw attention to context, qualitative research methodologies can be used to enhance the focus on experience and meaning made by athletes, mainly due to the emphasis on participants’ experiences, understandings, and perceptions, and the co-construction of findings (i.e., interaction between researcher and participant). Qualitative approaches can be used to shed light on the contextual experience (131), to examine athlete PWB in greater depth and detail (117). Future qualitative studies can begin to capture the range of sport-well-being experiences and more meaningful individualized understandings of well-being (132), while taking into account the unique psychosocial challenges faced by athletes in sport as they may play a role in determining important dimensions of athlete EWB. For example, researchers can employ grounded theory, reflexive thematic analysis, or interpretive phenomenological analysis to design studies that focus on understanding athletes' experiences with EWB. This is a significant area of research, where athlete driven understandings and conceptualizations are needed to truly comprehend “athlete EWB.”

Much of the existing research fails to take an individual approach to understanding the personal well-being experience (132). Individual approaches “emphasize the need to understand the domains in which well-being is experienced…there is a need for alternative qualitative approaches to study the relationship between sport and well-being that go beyond that of simply identifying and measuring relationships between varied concepts and focus more upon the lived experience of well-being” (132). In other words, well-being is contextually situated, and in order to understand athlete EWB research needs to explore “sport” and “EWB” together (i.e., sport-specific EWB) from individuals in that context (i.e., athletes). An examination into one's well-being needs to take in account “the individual” and “the context”. We can think about using qualitative methods to explore athlete EWB, asking different athletes what EWB in sport means to them. Within qualitative analysis there is the ability to interpret participants' experiences and meaning making within their psych-social world (133). Drawing attention to the sport context can help in better understanding *what* composes athlete EWB, as peoples' judgements (123) and understandings of their well-being are significantly related to the (personally) *important* contextual domains in one's life. For example, what are (potentially) important contextual components of sport/athlete EWB? In order to promote an environment that fosters athlete EWB, attention needs to be drawn to the athlete sport environment (134), and the contextual factors that could contribute to or disrupt athlete EWB. Future research into athletes’ personal experience of EWB in sport is needed to develop athlete driven descriptions of sport-specific EWB to capture a complete understanding of athlete EWB.

## Conclusion

There is growing research supporting the notion that well-being is valuable, not only because it is associated with “feeling good” but that it is central to increased productivity, improved overall health, longevity, resilience, personal growth, and quality of life (14, 30, 37, 52, 135, 136). This is also the case in the sport context; there is a growing body of literature surrounding athlete well-being and recognition of the “multilevel significance of well-being for athletes” health, development and performance as well as their lives beyond and after sport” (83). However, there are no actual strategies to enhance athlete well-being (137). Authors highlighted the inconsistent conceptualization of athlete EWB in the literature (137), as a potential reason for the lack of strategies to enhance athlete well-being.

Due to the conceptual opacity, inconsistent application, and mix and match measures of well-being in sport research, there is a call for theoretical sound and coherent well-being studies. Emerging research needs to operate from a distinct well-being tradition explicitly outlining a definition of well-being for the study, theoretically aligning measurements of well-being with the selected well-being perspective, and examining the contextual realm of sport-specific EWB. This is needed in order to move forward with supporting athletes' mental health and well-being, as there is a lack of consensus, clarity, and understanding of what comprises *athlete* EWB. This research can then help inform health professionals on the best practices to support athlete EWB, mitigating the potential for poor mental health.

## References

[1] Bennett EV Trainor LR Bundon AM Tremblay M Mannella S Crocker PRE. From “blessing in disguise” to “what do I do now?”: how Canadian olympic and paralympic hopefuls perceived, experienced, and coped with the postponement of the Tokyo 2020 games. Psychol Sport Exerc. (2022) 62:102246. 10.1016/j.psychsport.2022.102246

[2] Bundon A Trainor LR Bennett EV Tremblay MI Mannella S Crocker PRE. From minding the gap to widening the gap: paralympic athletes’ experiences of wellbeing during the postponement of the Tokyo 2020 games. Front Sports Act. Living. (2022) 4:921625. 10.3389/fspor.2022.921625 36091870 PMC9459162

[3] Fruchart E Rulence-Pâques P. Predicting sports performance from wellbeing: a mapping of professional athletes’, amateur athletes’ and non-athletes’ positions. Eur Rev Appl Psychol. (2022) 72(6):100793. 10.1016/j.erap.2022.100793

[4] Kouali D Hall C Divine A Pope P. Motivation and eudaimonic wellbeing in athletes: a self-determination theory perspective. Res Q Exerc Sport. (2022) 93(3):457–66. 10.1080/02701367.2020.1864259 34236282

[5] McLoughlin E Fletcher D Slavich GM Aronld R Moore LJ. Cumulative lifetime stress exposure, depression, anxiety, and well-being in elite athletes: a mixed-method study. Psychol Sport Exerc. (2021) 52:1018–23. 10.1016/j.psychsport.2020.101823 PMC7710337 33281503

[6] Moesch K Kenttä G Kleinert J Quignon-Fleur C Cecil S Bertollo M. FEPSAC Position statement: mental health disorders in elite athletes and models of service provision. Psychol Sport Exerc. (2018) 38:61–71. 10.1016/j.psychsport.2018.05.013

[7] Pankow K McHugh T-LF Mosewich AD Holt NL. Mental health protective factors among fourishing Canadian women university student-athletes. Psychol Sport Exerc. (2021) 52:101847. 10.1016/j.psychsport.2020.101847

[8] Puce L Okwen PM Yuh MN Akah Ndum Okwen G Pambe Miong RH Kong JD Well-being and quality of life in people with disabilities practicing sports, athletes with disabilities, and para-athletes: insights from a critical review of the literature. Front Psychol Movement Sci. (2023) 14:1071656. 10.3389/fpsyg.2023.1071656 PMC9945540 36844305

[9] Rice S Walton CC Gwyther K Purcell R. Mental health. In: Arnold R Fletcher D, editors. Stress, well-being and performance in sport. London: Routledge (2021). p. 167–87.

[10] Trainor LR Crocker PRE Bundon A Ferguson L. The rebalancing act: injured varsity women athletes’ experiences of global and sport psychological well-being. Psychol Sport Exerc. (2020) 49:101713. 10.1016/j.psychsport.2020.101713

[11] Trainor LR Bennett EV Bundon AM Tremblay M Mannella S Crocker PRE. Inescapable tensions: performance and/or psychological well-being in Olympic and paralympic athletes during sport disruption. Qual Res Sport Exerc Health. (2023) 15(5):601–18. 10.1080/2159676X.2023.2175899

[12] Uzzell KS Knight CJ Hill DM. Understanding and recognizing high performance swimmers’ well-being. Sport Exerc Perform Psychol. (2021) 11(1):12–27. 10.1037/spy0000284

[13] Deci EL Ryan RM. Hedonia, eudaimonia, and well-being: an introduction. J Happiness Stud. (2008) 9:1–11. 10.1007/s10902-006-9018-1

[14] Keyes CLM. The mental health continuum: from languishing to flourishing in life. J Health Soc Behav. (2002) 43:207–22. 10.2307/3090197 12096700

[15] Magyar JL Keyes CLM. Defining, measuring, and applying subjective well-being. In: Gallagher MW Lopez SJ, editors. Positive psychological assessment: A handbook of models and measures. Washington, DC: American Psychological Association (2019). p. 389–415. 10.1037/0000138-025

[16] Sharma-Brymer V Brymer E. Flourishing and eudaimonic well-being. Cham, Switzerland: Springer (2020).

[17] Tatarkiewicz W. Analysis of happiness. Martinus Nijhoff: The Hague, Netherlands (1976). 10.1007/978-94-010-1380-2

[18] Aristotle. McKeon R, editors. Introduction to aristotle. New York: The Modern Library (1947).

[19] Keyes CLM Shmotkin D Ryff C. Optimizing well-being: the empirical of two traditions. JPers Soc Development. (2002) 82:1007–2. 10.1037/0022-3514.82.6.1007 12051575

[20] Aristotle. In: Irwin T, editors. Nicomachean ethics. Indianapolis, iN: Hackett (1985).

[21] Waterman AS. The relevance of aristotle’s conception of eudaimonia for the psychological study of happiness. Theoretical Philos Psychol. (1990) 10:39–44. 10.1037/h0091489

[22] Huta V Waterman A. Eudaimonia and its distinction from hedonia: developing a classification and terminology for understanding conceptual and operational definitions. J Happiness Stud. (2014) 15:1425–56. 10.1007/s10902-013-9485-0

[23] Jayawickreme E Forgeard MJ Seligman MEP. The engine of well-being. Rev Gen Psychol. (2012) 16:327–42. 10.1037/a0027990

[24] Ryan RM Deci EL. On happiness and human potentials: a review of research on hedonic and eudaimonic well-being. Annu Rev Psychol. (2001) 52:141–66. 10.1146/annurev.psych.52.1.141 11148302

[25] Ryan RM Huta V Deci EL. Living well: a self-determination theory perspective on eudaimonia. J Happiness Stud. (2008) 9:139–70. 10.1007/s10902-006-9023-4

[26] Keyes CLM. Subjective well-being in mental health and human development research worldwide: an introduction. Soc Indic Res. (2006) 77:1–10. 10.1007/s11205-005-5550-3

[27] Nelson-Coffey K Schmitt J. Eudaimonia and flourishing. In: Friedman HS Markey CH, editors. Encyclopedia of mental health. Cambridge: Academic Press (2023). p. 821–7. 10.1016/B978-0-323-91497-0.00091-6.

[28] Thrash TM. The creation and curation of all things worthy: inspiration as vital force in persons and cultures. In: Elliot AJ, editors. Advances in motivation science. Cambridge: Elsevier Academic Press (2021). p. 181–244. 10.1016/bs.adms.2020.01.002

[29] Diener EL. Subjective well-being: the science of happiness and a proposal for a national index. Am Psychol. (2000) 55:34–43. 10.1037/0003-066X.55.1.34 11392863

[30] Diener E Lucas RE Oishi S. Advances and open questions in the science of subjective well-being. Collabra. (2018) 4(1):15. 10.1525/collabra.115 30637366 PMC6329388

[31] Diener EL Sapyta J Suh E. Subjective well-being is essential to well-being. Psychol Inq. (1998) 9:33–7. 10.1207/s15327965pli0901_3

[32] Diener EL. Guidelines for national indicators of subjective well-being and ill-being. Appl Res Qual Life. (2006) 1:151–7. 10.1007/s11482-006-9007-x

[33] Kahneman D Diener E Schwarz N. Well-being: The foundations of hedonic psychology. Russell Sage Foundation (1999).

[34] Ryff CD. Happiness is everything, or is it? Explorations on the meaning of psychological well-being. J Pers Soc Psychol. (1989) 57:1069–81. 10.1037/0022-3514.57.6.1069

[35] Ryff C Singer B. Know thyself and become what you are: a eudaimonic approach to psychological well-being. J Happiness Stud. (2008) 9:13–39. 10.1007/s10902-006-9019-0

[36] Waterman AS Schwartz SJ Zamboanga BL Ravert RD Williams MK Agocha VB The questionnaire for eudaimonic well-being: psychometric properties, demographic comparisons, and evidence of validity. J Posit Psychol. (2010) 5(1):41–61. 10.1080/17439760903435208 34326891 PMC8317967

[37] Ryff CD Singer BH Love GD. Positive health: connecting well-being with biology. Philos Trans Roy Soc London B. (2004) 359:1383–94. 10.1098/rstb.2004.1521 15347530 PMC1693417

[38] Waterman AS. Two conceptions of happiness: contrasts of personal expressiveness (eudaimonia) and hedonic enjoyment. J Pers Soc Psychol. (1993) 64:678–91. 10.1037/0022-3514.64.4.678

[39] Huta V Ryan RM. Pursuing pleasure or virtue: the differential and overlapping well-being benefits of hedonic and eudaimonic motives. J Happiness Stud. (2010) 11:735–62. 10.1007/s10902-009-9171-4

[40] Bauer JJ McAdams DP Sakaeda A. Interpreting the good life: growth memories in the lives of mature, happy people. J Pers Soc Psychol. (2005) 88(1):203–17. 10.1037/0022-3514.88.1.203 15631585

[41] Heintzelman SJ. Eudaimonia in the contemporary science of subjective well-being: psychological well-being, self-determination, and meaning in life. In: Diener E Oishi S Tay L, editors. Handbook of well-being. Salt Lake City, UT: DEF (2018). p. 145–57.

[42] Martela F Sheldon KM. Clarifying the concept of well-being: psychological need-satisfaction as the common core connecting eudaimonic and subjective well-being. Rev Gen Psychol. (2019) 23(4):458–74. 10.1177/1089268019880886

[43] Cooke PJ Melchert TP Connor K. Measuring wellbeing: a review of instruments. Couns Psychol. (2016) 44:730–57. 10.1177/0011000016633507

[44] Seligman MEP. Authentic happiness. New York, NY: Free Press (2002).

[45] Huta V. Pursuing eudaimonia versus hedonia: Distinctions, similarities, and relationships. In: Waterman AS, editor. The best within us: Positive psychology perspectives on eudaimonia. Washington, DC: American Psychological Association (2013). p. 139–58. 10.1037/14092-008

[46] Bauer JJ McAdams DP Pals J. Narrative identity and eudaimonic well-being. J Happiness Stud. (2008) 9:81–104. 10.1007/s10902-006-9021-6

[47] Fletcher D Hanton S Mellalieu SD. An organizational stress review: conceptual and theoretical issues in competitive sport. In: Hanton S Mellalieu SD, editors. Literature reviews in sport psychology. Hauppauge, NY: Nova Science Publishers (2006). p. 321–74.

[48] Vittersø J. Feelings, meanings, and optimal functioning: some distinctions between hedonic and eudaimonic well-being. In: Waterman AS, editor. The best within us: Positive psychology perspectives on eudaimonia. Washington, DC: American Psychological Association (2013). p. 39–55. 10.1037/14092-003

[49] Delle Fave A Brdar I Freire T Vella-Brodrick D Wissing M. The eudaimonic and hedonic components of happiness: qualitative and quantitative findings. Soc Indic Res. (2011) 100(2):185–207. 10.1007/s11205-010-9632-5

[50] Fowers BJ. An aristotelian framework for the human good. J Theor Philos Psychol. (2012) 32:10–23. 10.1037/a0025820

[51] Steger MF Shin J-Y Shim Y Fitch-Martin A. Is meaning in life a flagship indicator of well-being? In: Waterman A, editors. Eudaimonia. Washington, DC: APA Press (2013). p. 159–82.

[52] Huppert FA So TTC. Flourishing across Europe: application of a new conceptual framework for defining well-being. Soc Indic Res. (2013) 110:837–61. 10.1007/s11205-011-9966-7 23329863 PMC3545194

[53] Diener E Wirtz D Tov W Kim-Prieto C Choi DW Oishi S New well-being measures: short scales to assess flourishing and positive and negative feelings. Soc Indic Res. (2010) 97:143–56. 10.1007/s11205-009-9493-y

[54] Joshanloo M. Revisiting the empirical distinction between hedonic and eudaimonic aspects of well-being using exploratory structural equation modeling. J Happiness Stud. (2016) 17:2023–36. 10.1007/s10902-015-9683-z

[55] Lee E Carey T. Eudaimonic well-being as a core concept of positive functioning. MindPad. (2013) 2(1):17–20.

[56] Westerhof GJ Keyes CL. Mental illness and mental health: the two continua model across the lifespan. J Adult Dev. (2010) 17(2):110–9. 10.1007/s10804-009-9082-y 20502508 PMC2866965

[57] Huta V. An overview of hedonic and eudaimonic well-being concepts. In: Reinecke L Oliver MB, editors. The routledge handbook of media use and well-being: international perspectives on theory and research on positive media effects. New York: Routledge (2017). p. 14–33.

[58] Keyes CL. Mental illness and/or mental health? Investigating axioms of the complete state model of health. J Consult Clin Psychol. (2005) 73(3):539–48. 10.1037/0022-006X.73.3.539 15982151

[59] McMahan EA Estes D. Hedonic versus eudaimonic conceptions of well-being. Soc Indic Res. (2011) 103:93–108. 10.1007/s11205-010-9698-0

[60] Keyes CLM Annas J. Feeling good and functioning well: distinctive concepts in ancient philosophy and contemporary science. J Posit Psychol. (2009) 4(3):197–201. 10.1080/17439760902844228

[61] MacDougall H O’Halloran P Shields N Sherry E. Comparing the well-being of para and Olympic sport athletes: a systematic review. Adapt Phys Act. (2015) 32:256–76. 10.1123/APAQ.2014-0168 26113553

[62] Gould D Dieffenbach K Moffett A. Psychological characteristics and their development in Olympic champions. J Appl Sport Psychol. (2002) 14:172–204. 10.1080/10413200290103482

[63] Purcell R Gwyther K Rice SM. Mental health in elite athletes: increased awareness requires an early intervention framework to respond to athlete needs. Sports Med Open. (2019) 5:46. 10.1186/s40798-019-0220-1 31781988 PMC6883009

[64] Rice SM Purcell R De Silva S Mawren D McGorry PD Parker AG. The mental health of elite athletes: a narrative systematic review. Sports Med. (2016) 46(9):1333–53. 10.1007/s40279-016-0492-2 26896951 PMC4996886

[65] Reardon CL Hainline B Aron CM Baron D Baum AL Bindra A Mental health in elite athletes: international olympic committee consensus statement (2019). Br J Sports Med. (2019) 53(11):667–99. 10.1136/bjsports-2019-100715 31097450

[66] Hughes L Leavey G. Setting the bar: athletes and vulnerability to mental illness. Br J Psychiatry. (2012) 200:95–6. 10.1192/bjp.bp.111.095976 22297587

[67] Foskett R Longstaff F. The mental health of elite athletes in the United Kingdom. J Sci Med Sport. (2018) 21(8):765–70. 10.1016/j.jsams.2017.11.016 29289498

[68] Gorczynski P Coyle M Gibson K. Depressive symptoms in high-performance athletes and non-athletes: a comparative meta-analysis. Br J Sports Med. (2017) 51(18):1348–54. 10.1136/bjsports-2016-096455 28254747

[69] Gulliver A Griffiths KM Christensen H. Barriers and facilitators to mental health help-seeking for young elite athletes: a qualitative study. BMC Psychiatry. (2012) 12:157. 10.1186/1471-244X-12-157 23009161 PMC3514142

[70] Reardon CL Factor RM. Sport psychiatry: a systematic review of diagnosis and medical treatment of mental illness in athletes. Sport Med. (2010) 40:961–80. 10.2165/11536580-000000000-00000 20942511

[71] Keyes CLM Dhingra SS Simoes EJ. Change in level of positive mental health as a predictor of future risk of mental illness. Am J Public Health. (2010) 100:2366–71. 10.2105/AJPH.2010.192245 20966364 PMC2978199

[72] Didymus F Rumbold J Staff H. Promoting and protecting coach psychological well-being and performance. Prof Adv Sports Coach. (2018):261–76. 10.4324/9781351210980-17

[73] Hill DM Brown G Lambert T-L Mackintosh K Knight C Gorczynski P. Factors perceived to affect the wellbeing and mental health of coaches and practitioners working within elite sport. Sport Exerc Perform Psychol. (2021) 10(4):504–18. 10.1037/spy0000263

[74] Norris LA Didymus FF Kaiseler M. Stressors, coping, and well-being among sports coaches: a systematic review. Psychol Sport Exerc. (2017) 33:93–112. 10.1016/j.psychsport.2017.08.005

[75] Potts AJ Didymus FF Kaiseler M. Bringing sports coaches’ experiences of primary appraisals and psychological well-being to life using composite vignettes. Qual Res Sport Exerc Health. (2021) 14(5):778–95. 10.1080/2159676X.2021.1948913

[76] Simpson RA Didymus FF Williams TL. Interpersonal psychological wellbeing among coach-athlete-sport psychology practitioner triads. Psychol Sport Exerc. (2023) 67:102435. 10.1016/j.psychsport.2023.102435 37665888

[77] Kim M Kim YD Lee HW. It is time to consider athletes’ well-being and performance satisfaction: the roles of authentic leadership and psychological capital. Sport Manag Rev. (2020) 23(5):964–77. 10.1016/j.smr.2019.12.008

[78] Wicker P Thormann TF. Well-being of sport club members: the role of pro-environmental behavior in sport and clubs’ environmental quality. Sport Manag Rev. (2022) 25(4):567–88. 10.1080/14413523.2021.1991688

[79] Simpson RAC Didymus FF Williams TL. Organizational stress and well-being in competitive sport: a systematic review. Int Rev Sport Exerc Psychol. (2021):1–29. 10.1080/1750984X.2021.1975305

[80] Kouali D Hall C Deck S. Examining the effectiveness of an imagery intervention in enhancing athletes’ eudaimonic well-being. J Imagery Res Sport Phys Act. (2020) 15(1):20200003. 10.1515/jirspa-2020-0003

[81] Macdougall H O'Holloran P Sherry E Shields N. Needs and strengths of Australian para athletes: identifying the subjective, psychological, social, and physical health and well-being. Sport Psychol. (2016) 30:1–12. 10.1123/tsp.2015-0006

[82] Macdougall H O'Halloran P Sherry E Shields N. A pilot randomised controlled trial to enhance well-being and performance of athletes in para sports. Eur J Adapted Phys Act. (2019) 12:1–19. 10.5507/euj.2019.006

[83] Purcell R Henderson J Tamminen KA Frost J Gwyther K Kerr G Starting young to protect elite athletes' mental health. Br J Sports Med. (2023) 57(8):439–40. 10.1136/bjsports-2022-106352 36796858 PMC10086277

[84] Campbell N Brady A Tincknill-Smith A. Developing and supporting athlete well-being: person first, athlete second. London: Routledge (2021). doi: 10.4324/9780429287923.

[85] Giles S Fletcher D Arnold R Ashfield A Harrison J. Measuring well-being in sport performers: where are we now and how do we progress? Sports Med. (2020) 50:1255–70. 10.1007/s40279-020-01274-z 32103451 PMC7305091

[86] Quested E Duda JL. Exploring the social-environmental determinants of well- and ill-being in dancers: a test of basic needs theory. J Sport Exerc Psychol. (2010) 32:39–60. 10.1123/jsep.32.1.39 20167951

[87] Clement D Arvinen-Barrow M Fetty T. Psychosocial responses during different phases of sport-injury rehabilitation: a qualitative analysis. J Athl Train. (2015) 50:95–104. 10.4085/1062-6050-49.3.52 25322346 PMC4299742

[88] Orlowski J Wicker P. Putting a price tag on healthy behavior: the monetary value of sports participation to individuals. Appl Res Qual Life. (2018) 13:479–99. 10.1007/s11482-017-9536-5

[89] Wicker P Dallmeyer S Breuer C. Elite athlete well-being: the role of socioeconomic factors and comparisons with the resident population. J Sport Manag. (2020) 34:341–53. 10.1123/jsm.2019-0365

[90] Chatzisarantis NL Hagger MS. The moral worth of sport reconsidered: contributions of recreational sport and competitive sport to life aspirations and psychological well-being. J Sports Sci. (2007) 25:1047–56. 10.1080/02640410600959954 17497406

[91] Mosewich A Kowalski K Crocker PRE. Managing injury and other setbacks in sport: experiences of (and resources for) high performance women athletes. Qual Res Sport Exerc Health. (2014) 6(2):182–204. 10.1080/2159676X.2013.766810

[92] Fletcher D Sarkar M. A grounded theory of psychological resilience in Olympic champions. Psychol Sport Exerc. (2012) 13:669–78. 10.1016/j.psychsport.2012.04.007

[93] Steffen K Soligard T Engebretsen L. The IOC’s endeavor to protect the health of the athlete continues. Br J Sports Med. (2011) 45:551–2. 10.1136/bjsports-2011-090070 21714166

[94] Amorose AJ Anderson-Butcher D Cooper J. Predicting changes in athletes’ well-being from changes in need satisfaction over the course of a competitive season. Res Q Exerc Sport. (2009) 80:386–92. 10.1080/02701367.2009.10599575 19650406

[95] Kouali D Hall C Pope P. Measuring eudaimonic wellbeing in sport: validation of the eudaimonic wellbeing in sport scale. Int J Wellbeing. (2020) 10:93–106. 10.5502/ijw.v10i1.776

[96] Lundqvist C Sandin F. Well-being in elite sport: dimensions of hedonic and eudaimonic well-being among elite orienteers. Sport Psychol. (2014) 28:245–54. 10.1123/tsp.2013-0024

[97] Reinboth M Duda JL Ntoumanis N. Dimensions of coaching behavior, need satisfaction, and the psychological and physical welfare of young athletes. J Motiv Emot. (2004) 28:297–313. 10.1023/B:MOEM.0000040156.81924.b8

[98] Adie J Duda J Ntoumanis N. Achievement goals, competition appraisals, and the psychological and emotional welfare of sports participants. J Sport Exerc Psychol. (2008) 30:302–22. 10.1123/jsep.30.3.302 18648108

[99] Blanchard C Amoit C Pereault S Vallerand R Provencher P. Cohesiveness, coach’s interpersonal style and psychological needs: their effects on self-determination and athletes’ subjective well-being. Psychol Sport Exerc. (2009) 10:546–51. 10.1016/j.psychsport.2009.02.005

[100] Houltberg B Wang K Qi W Nelson C. Self-narrative profiles of elite athletes and comparisons of psychological well-being. Res Q Exerc Sport. (2018) 89(3):354–60. 10.1080/02701367.2018.1481919 29985771

[101] Mack D Gunnel K Gilchrist J Kowalski K Crocker PRE. Health-enhancing physical activity: associations with markers of well-being. Appl. Psychol Health Well-Being. (2012) 4(2):127–50. 10.1111/j.1758-0854.2012.01065.x 26286974

[102] Podlog L Lochbaum M Stevens T. Need satisfaction, well-being, and perceived return-to-sport outcomes among injured athletes. J Appl Sport Psychol. (2010) 22:167–82. 10.1080/10413201003664665

[103] Smith AL Ntoumanis N Duda JL Vansteenkiste M. Goal striving, coping, and well-being: a prospective investigation of the self-concordance model in sport. J Sport Exerc Psychol. (2011) 33:124–45. 10.1123/jsep.33.1.124 21451174

[104] Vallerand RJ Salvy S-J Mageau GA Elliot AJ Denis PL Grouzet FME On the role of passion in performance. J Pers. (2007) 75:505–33. 10.1111/j.1467-6494.2007.00447.x 17489890

[105] Vallerand RJ Pelletier LG Koestner R. Reflections on self-determination theory. Can Psychol. (2008) 49:257–62. 10.1037/a0012804

[106] Verner-Filion J Vallarand R Amiot C Mocanu I. Two roads from passion to sport performance and psychological well-being: the mediating role of need satisfaction, deliberate practice and achievement goals. Psychol Sport Exerc. (2017) 30:19–29. 10.1016/j.psychsport.2017.01.009

[107] Ryff C Keyes L. The structure of psychological well-being revisited. J Pers Soc Psychol. (1995) 69:719–27. 10.1037/0022-3514.69.4.719 7473027

[108] Ferguson L Kowalski K Mack D Wilson P Crocker PRE. Women’s health-enhancing physical activity and eudaimonic well-being. Res Q Exerc Sport. (2012) 83(3):451–63. 10.1080/02701367.2012.10599880 22978195

[109] Cowen EL. In pursuit of wellness. Am Psychol. (1991) 46:404–8. 10.1037/0003-066X.46.4.404

[110] Keyes CLM. Promoting and protecting mental health as flourishing: a complementary strategy for improving national mental health. Am Psychol. (2007) 62:95–108. 10.1037/0003-066X.62.2.95 17324035

[111] Martela F Sheldon KM. Clarifying the concept of well-being: psychological need-satisfaction as the common core connecting eudaimonic and subjective well-being. Rev Gen Psychol. (2019) 23(4):458–74. 10.1177/1089268019880886

[112] Vittersø J. Feelings, meanings, and optimal functioning: some distinctions between hedonic and eudaimonic well-being. In: Waterman A, editors. The best within US: positive psychology perspectives on eudaimonia. American Psychological Association (2013). p. 39–55.

[113] Watson D Clark LA Tellegen A. Development and validation of brief measures of positive and negative affect: the PANAS scales. J Pers Soc Psychol. (1988) 54(6):1063. 10.1037/0022-3514.54.6.1063 3397865

[114] Diener E Emmons RA Larsen RJ Griffin S. The satisfaction with life scale. J Pers Assess. (1985) 49:71–5. 10.1207/s15327752jpa4901_13 16367493

[115] Ryan RM Frederick CM. On energy, personality and health: subjective vitality as a dynamic reflection of well-being. J Pers. (1997) 65:529–65. 10.1111/j.1467-6494.1997.tb00326.x 9327588

[116] Tennant R Hiller L Fishwick R Platt S Joseph S Weich S The warwick-Edinburgh mental well-being scale (WEMWBS): development and UK validation. Health Qual Life Outcome. (2007) 5:1–13. 10.1186/1477-7525-5-63 PMC2222612 18042300

[117] Lundqvist C. Well-being in competitive sports—the feel-good factor? A review of conceptual considerations of well-being. Int Rev Sport Exerc Psychol. (2011) 4:109–27. 10.1080/1750984X.2011.584067

[118] Kouali D Hall C Pope P. Examining an adapted version of Ryff’s scales of psychological well-being in sport. J Health Phys Act. (2018) 10(4):213–25. 10.29359/BJHPA.10.4.20

[119] Mack D Wilson P Oster K Kowalski K Crocker PRE Sylvester B. Wellbeing in volleyball players: examining the contributions of independent and balanced psychological need satisfaction. J Psychol Sport Exerc. (2011) 12:533–9. 10.1016/j.psychsport.2011.05.006

[120] Ferguson L Kowalski K Mack D Sabiston C. Exploring self-compassion and eudaimonic well-being in young women athletes. J Sport Exerc Psychol. (2014) 36:203–16. 10.1123/jsep.2013-0096 24686956

[121] Mirehie M Gibson H. Women’s participation in snow-sports and sense of well-being: a positive psychology approach. J Leis Res. (2020) 51(4):397–415. 10.1080/00222216.2019.1702485

[122] Foster BJ Chow GM. Development of the sport mental health Continuum—short form (sport MHC-SF). J Clin Sport Psychol. (2019) 13:593–608. 10.1123/jcsp.2017-0057

[123] Diener EL Lucas R Scollon S. The evolving concept of subjective well-being: the multifaceted nature of happiness. Adv Cell Aging Gerontol. (2003) 15:187–219. 10.1016/S1566-3124(03)15007-9

[124] Tracey J Elcombe T. A lifetime of healthy meaningful movement: have we forgotten the athletes? Quest. (2004) 56:241–60. 10.1080/00336297.2004.10491825

[125] Schwarts N Stack F. Reports of subjective well-being: judgment processes and their methodological implications. In: Kahneman D Diener E Schwartz N, editors. Foundation of hedonic psychology: scientific perspectives on enjoyment and suffering. New York: Russell Sage Foundation (1999). p. 61–84.

[126] Page KM Vella-Brodrick DA. The “what”, “why” and “how” of employee well-being: a new model. Soc Indic Res. (2009) 90:441–58. 10.1007/s11205-008-9270-3

[127] OECD. OECD Guidelines on measuring subjective well-being. European Union: OECD Publishing (2013). doi: 10.1787/9789264191655-en 24600748

[128] Lehnert K Sudeck G Conzelmann A. Subjective well-being and exercise in the second half of life: a critical review of theoretical approaches. Eur Rev Aging Phys Act. (2012) 9(2):1–16. 10.1007/s11556-012-0095-3

[129] Huppert FA Whittington JE. Evidence for the independence of positive and negative well-being: implications for quality of life assessment. Br J Health Psychol. (2003) 8:107–22. 10.1348/135910703762879246 12643820

[130] Goodman FR Disabato DJ Kashdan TB Kaufman SB. Measuring well-being: a comparison of subjective well-being and PERMA. J Posit Psychol. (2018) 13:321–32. 10.1080/17439760.2017.1388434

[131] Braun V Clarke V. Can I use TA? Should I use TA? Should I not use TA? Comparing reflexive thematic analysis and other pattern-based qualitative analytic approaches. Couns Psychother Res. (2021) 21:37–47. 10.1002/capr.12360

[132] Mayoh J Jones I. Making well-being an experiential possibility: the roles of sport. Qual Res Sport Exerc Health. (2015) 7(2):235–53. 10.1080/2159676X.2014.893901

[133] Braun V Clarke V. Conceptual and design thinking for thematic analysis. Qual Psychol. (2022) 9(1):3–26. 10.1037/qup0000196

[134] Henriksen K Schinke R McCann S Durand-Bush N Moesch K Parham WD Athlete mental health in the Olympic/ paralympic quadrennium: a multi-societal consensus statement. Int J Sport Exerc Psychol. (2020) 18(3):391–408. 10.1080/1612197X.2020.1746379

[135] De Neve JE Krekel C Ward G. Work and well-being: a global perspective. In: Sachs JD Bin Bashir A De Neve JE Durand M Diener E Helliwell JF Layard R Seligman M, editors. Global happiness: policy report. New York: Sustainable Development Solutions Network (2018). p. 74–128.

[136] Lyubomirsky S King LA Diener E. The benefits of frequent positive affect: does happiness lead to success? Psychol Bull. (2005) 131:803–55. 10.1037/0033-2909.131.6.803 16351326

[137] Chambers TP Harangozo G Mallett CJ. Supporting elite athletes in a new age: experiences of personal excellence advisers within Australia’s high-performance sporting environment. Qual Res Sport Exerc Health. (2019) 11(5):650–70. 10.1080/2159676X.2019.1605404

